# Supplementary material for: Robust point-process Granger causality analysis in presence of exogenous temporal modulations and trial-by-trial variability in spike trains
Source: PLoS Comput Biol. 2021 Jan 25;17(1):e1007675. doi: 10.1371/journal.pcbi.1007675 (PMC7861554; doi:10.1371/journal.pcbi.1007675)
Supplement: Attachment — Submitted filename: ploscb_granger.pdf [file pcbi.1007675.s004.pdf]

Review of PCOMPBIOL-D-20-00115

This manuscript presents a valid contribution to current studies on Granger causality and is suitable to PLOS Computational Biology. I therefore recommend its acceptance. I believe, however, that some essential technical points need to be clarified, possibly amended. And some explanations could be made more precise.

1. The examples (mostly summarized in Figs 2, 3) demonstrating the model emphasize the reduction of false positives as compared with Kim et al. What about false negatives, especially with weak exogenous input? Some readers will fear that the model may underestimate Granger-causal connections that are 'truly' there. I recommend to include the results for networks like the ones of Figs 2 and 3 but without exogenous input, or with a much weaker one. If the authors have no time to perform such additional simulations, can they at least explain, in the Introduction and Discussion, that the model hasn't been tested for false negatives with weak exogenous inputs?

2. The reported percentages for the results of the simulation of Fig. 4 are unclear (lines 136–144). For example, what do the 42% and 4.2% proportions of false positives refer to? are they over the total number of connections × trials, 16 × 100? or over the trials with at least one wrong connection? Same holds for the 85% and 88%. Analogous questions arise for the simulation described on lines 216–224.

3. It's surprising and interesting that the fitting of the model of eq. (4) doesn't end up setting the γ-terms to zero and using the exogenous α-term only, which offers a large amount of degrees of freedom. Were any regularizers or bounds used in the fitting? Or is this the effect of Akaike's criterion? in the latter case, how large was N_i thus found? It'd be useful if the details of the fitting, only generically mentioned on lines 361–369, were reported in more detail.

4. I don't understand how the model of eq. (6) can be fitted at all. For this model, one set of trials (such as the 40 trials of Fig. 4) constitutes *one* data point – surely so for the fitting of the A-parameters. In the example of lines 212–222 the model therefore seems to be fitted with one data point. From a 'frequentist' viewpoint such fit hardly seems possible. From a Bayesian viewpoint it is possible: the prior information will simply play a dominant role in the fitting. But this prior information should then

be explicitly stated. How are we to interpret the fitting procedure in the present case?

5. Important question: why the linear dependence of the two models, eqs (4) and (6)? What happens with different functional forms? How uncertain are our conclusions about causal links, when we consider our uncertainty about the structure of the model?

The Monte Carlo simulations of lines 136–144 and 212–222 were generated by a process having essentially the same form as the model, as explained on lines 387–399. So they 'validate' the model only in the sense of ascertaining the absence of bugs in its numerical implementation. They don't validate the model in the sense of its appropriateness for real data. No such validation can in fact exist: it would require knowledge of the real-data 'ground truth' – and if we knew the ground truth we wouldn't be here making models. Such validations, in this sense, are always circular; their purpose is only to give a pat on the back.

It is known (Draper 1995; Chatfield 1995) that the uncertainty in the model itself (also called structural uncertainty) is 'likely to be more serious than other sources of uncertainty' (Chatfield), 'leading to inaccurate scientific summaries and overconfident decisions that do not incorporate sufficient hedging against uncertainty' (Draper). It should preferably be assessed – and this is indeed possible, see the cited papers – or at the very least pointed out and discussed.

What are the results of the models on data generated by a different, synthetic but more realistic model, say a leak-integrate-and-fire one?

6. I appreciate the mathematical explanations (e.g. around lines 78 and 95), in terms of number of parameters, of why Kim et al.'s method can infer misleading Granger-causal links, whereas the proposed methods can avoid this. But the deeper and more general reason – connected with the very notion of 'causality' – surprisingly isn't mentioned. And this reason is important to understand what we're doing with our modelling.

Phenomena or situations $B_1, B_2, \dots$ are considered the causes of event A if, roughly speaking, (i) all B_i precede A in time, (ii) knowledge of the B_i changes our uncertainty about A, (iii) knowledge of other phenomena $C_1, C_2, \dots$ preceding A, besides the B_i, doesn't change our uncertainty about A. The last two conditions are expressed as

$$\mathrm{P}(A \mid I) \neq \mathrm{P}(A \mid B_1, B_2, \dots, I) = \mathrm{P}(A \mid B_1, B_2, \dots; C_1, C_2, \dots, I) \quad \forall C_i \;, \quad (1)$$

where I represents contextual knowledge necessary to define the problem.

Such a generic definition appears to be more or less agreed upon by many studies, from Freedman's (1991; 1997; 2004; 2006) to Pearl's (2009; 2003; 2004; 2018), from Simon's (1954), Good's (1961a,b), or Toda's (1977) to Valdes-Sosa et al.'s (2011). A very interesting perspective is given by Lindley and Novick (1981). The invariance represented by the second equality expresses the 'robustness' of causal links, when compared with non-causal correlations. Hence the testing of such robustness by interventions, as opposed to observations (Pearl & Mackenzie 2018 ch. 3, explain this very tersely in my opinion – even if one doesn't wholly agree with their whole work). The numerical values of these conditional probabilities can be compared in various ways (means, variance, tests, etc.).

In Granger's (1969 § 5) binary study, B_1 is the history of previous events of the same kind as A, the term C_1 is the history of a different kind of events, and others $B_2, C_2, \dots$ are *assumed not* to exist. So he can simply compare the conditional probabilities $\mathrm{P}(A \mid B_1, I)$ and $\mathrm{P}(A \mid B_1; C_1, I)$ above (ibid. p. 429) to ascertain whether C_1 is or isn't a cause of A, besides B_1.

If we know that other phenomena B_2 or C_2, such as extra-network inputs in a neural network, *are* present, we can't use Granger's binary case; that'd be a misapplication. The point is not that it can give 'wrong' results. The point is that whatever results it gives are meaningless, because the conditions for the definition and quantification of causal links are not met. For this reason Kim et al.'s method would be misapplied if used for a non-isolated neural network, and one needs the method proposed in the present manuscript (putting aside problems with model uncertainty, point 5 above).

7. Generalized linear models constitute an infinite-dimensional family of parametric models. It's extremely vague to say that we're using a generalized linear model. Laplace (1809–1810) could have said so too, since he used normal distributions. Saying so could be informative when we imply the existence and role of some sufficient statistics. But no such connection is made in this manuscript. I'd appreciate if at least the link function were specified when mentioning a general linear model.

8. How is 'information flow' (e.g. line 27) defined? what is it exactly?

9. Why is the difference of 85% vs 88% of G-ETM vs Kim (line 142) not significant, but the similar difference of 97% vs 92% of G-ETM vs G-ETMV is?

10. Figure 3 E, plot in 2nd row, 1st column: the blue curve (estimated interaction) seems to extend outside the plot range. How much? Can the plot range be fixed, to include the whole blue and black curves?

11. Figure 7 B, scatter-plot: the points are so many that they overlap, masking their high-density region. I recommend subsampling them so that readers can compare the red line and the density peak in the scatter-plot.

12. All plots are extremely small. Many readers print articles to read them; they'd be thankful to have larger, decipherable plots.

13. Please see the recent *official* statements by the American Statistical Association (ASA 2016; 2019):

> It is time to stop using the term "statistically significant" entirely. Nor should variants such as "significantly different," "$p < 0.05$," and "nonsignificant" survive, whether expressed in words, by asterisks in a table, or in some other way. [. . .]
>
> Where p-values are used, they should be reported as continuous quantities (e.g., $p = 0.08$). (ASA 2019 § 2)

I therefore recommend that the authors explicitly state the p-values they find, rather than reporting 'significant' vs 'not-significant'.

14. I genuinely thank the authors for limiting the number of acronyms. It was pleasant to read the manuscript without having to continuously jump back and forth trying to find where an unmemorizable random string of letters was defined. I recommend that the two models' acronyms be spelled out directly on lines 38–40.

The manuscript is well written and reads smoothly. It would be practical if eq. (4) were somehow written in full before line 101, where the γ-parameters are mentioned, or at least if these parameters were explained somewhat more around that line.

On line 27: 'the brain state is perturbed by an event' – can we really speak of 'perturbations', as if it was a stationary state? I think that the message conveyed around that line is valid even without speaking of 'perturbations'.

There are commas placed in strange, ungrammatical positions here and there; for example:

'the brain state, is perturbed' (line 27),

'because, the GLM' (line 77),

‘This means, that’ (line 123),
‘this happened because, trial-by-trial’ (line 191).

Line 48: it took me some time to understand that ‘endogenous, temporal’ weren’t adjectives of ‘modulations’. I’d recommend changing this sentence to ‘we will denote temporal modulations [. . .] as *endogenous*, and temporal modulations [. . .] as *exogenous*’.

In summary: some of the points above may appear very critical. But that’s because I consider (as stated earlier) this work to be a valid contribution. Especially so in view of point 6, which this work addresses directly. For this reason I’d like the points above to be clarified. There’s also another important reason. I’m generally concerned for master’s and PhD students, who often learn concepts and methods through actual research, perusing works like the present one. Young researchers too often end up learning incorrect or confused ideas, gathered from unclear literature. They trust it because it’s peer-reviewed. We owe them clarity, precision, honesty, and a glimpse of the bigger picture.

Some of the points above mention additional simulations or calculations. I’m not requesting the authors to do them – one can’t do everything, and other researchers or the authors themselves can address them in future works, as further developments of the present milestone. But I ask the authors to address and discuss those points at least verbally; I’m sure such discussions would benefit the manuscript and make it more robust.

References

ASA (American Statistical Association) (2016): *ASA statement on statistical significance and p-values*. Am. Stat. **70**^{2}, 131–133. Ed. by R. L. Wasserstein. See also introductory editorial in Wasserstein, Lazar (2016) and discussion in Greenland, Senn, Rothman, Carlin, Poole, Goodman, Altman, Altman, et al. (2016).

— (2019): *Moving to a world beyond “$p < 0.05$”*. Am. Stat. **73**^{S1}, 1–19. Ed. by R. L. Wasserstein, A. L. Schirm, N. A. Lazar.

Aykaç, A., Brumat, C., eds. (1979): *New Developments in the Applications of Bayesian Methods*, 2nd pr. (North-Holland, Amsterdam). First publ. 1977.

Berk, R. A., Blalock Jr., H. M., Mason, W. M., Freedman, D. A. (1991): *Toward a methodology for mere mortals. Are there really any constructive alternatives to causal modeling? Freedman is right as far as he goes, but there is more, and it’s worse. Statisticians could help. Rejoinder*. Sociol. Methodol. **21**, 315–358. See Freedman (1991).

Chatfield, C. (1995): *Model uncertainty, data mining and statistical inference*. J. Roy. Stat. Soc. A **158**^{3}, 419–444. See also discussion in Copas, Davies, Hand, Lunneborg, Ehrenberg, Gilmour, Draper, Green, et al. (1995).

Copas, J. B., Davies, N., Hand, D. J., Lunneborg, C. E., Ehrenberg, A. S., Gilmour, S. G., Draper, D., Green, P. J., et al. (1995): *Discussion of the paper by Chatfield [Model uncertainty, data mining and statistical inference]*. J. Roy. Stat. Soc. A **158**[3], 444–466. See Chatfield (1995).

Draper, D. (1995): *Assessment and propagation of model uncertainty*. J. Roy. Stat. Soc. B **57**[1], 45–70. See also discussion and reply in Spiegelhalter, Grieve, Lindley, Lindley, Copas, Cairns, Chatfield, Box, et al. (1995). https://classes.soe.ucsc.edu/ams206/Winter05/draper.pdf.

Fienberg, S. E., Haviland, A. M., Heckerman, D., Shachter, R., Kadane, J. B., Moral, S., Pearl, J. (2003): *[Statistics and causal inference: a review] discussion and rejoinder*. Test **12**[2], 319–345. See Pearl (2003).

Freedman, D. (1997): *From association to causation via regression*. Adv. Appl. Math. **18**[1], 59–110.

Freedman, D. A. (1991): *Statistical models and shoe leather*. Sociol. Methodol. **21**, 291–313. See also Berk, Blalock, Mason, Freedman (1991). http://psychology.okstate.edu/faculty/jgrice/psyc5314/Freedman_1991A.pdf.

— (2004): *Graphical models for causation, and the identification problem*. Eval. Rev. **28**[4], 267–293.

— (2006): *Statistical models for causation: what inferential leverage do they provide?* Eval. Rev. **30**[6], 691–713. https://www.stat.berkeley.edu/~census/oxcauser.pdf.

Good, I. J. (1961a): *A causal calculus (I)*. Brit. J. Phil. Sci. **XI**[44], 305–318. See also Good (1961b).

— (1961b): *A causal calculus (II)*. Brit. J. Phil. Sci. **XII**[45], 43–51. See also Good (1961a).

Granger, C. W. J. (1969): *Investigating causal relations by econometric models and cross-spectral methods*. Econometrica **37**[3], 424–438.

Greenland, S., Senn, S. J., Rothman, K. J., Carlin, J. B., Poole, C., Goodman, S. N., Altman, D. G., Altman, N. S., et al. (2016): *Online supplement and discussion: ASA statement on statistical significance and p-values*. Am. Stat. **70**[2], 129. http://www.tandfonline.com/doi/suppl/10.1080/00031305.2016.1154108. See ASA (2016) and Wasserstein, Lazar (2016).

Halpern, J. Y., Pearl, J. (2005a): *Causes and explanations: a structural-model approach. Part I: causes*. Brit. J. Phil. Sci. **56**[4], 843–887. See also Halpern, Pearl (2005b).

— (2005b): *Causes and explanations: a structural-model approach. Part II: explanations*. Brit. J. Phil. Sci. **56**[4], 889–911. See also Halpern, Pearl (2005a).

Laplace (Marquis de), P. S. (1809–1810): *Mémoire sur les approximations des formules qui sont fonctions de très grands nombres et sur leur application aux probabilités*. Mém. cl. sci. math. phys. Inst. Fr. **X**, 353–415, 559–565. http://gallica.bnf.fr/ark:/12148/bpt6k77600r/f306.

Lindley, D. V., Novick, M. R. (1981): *The role of exchangeability in inference*. Ann. Stat. **9**[1], 45–58.

Pearl, J. (2003): *Statistics and causal inference: a review*. Test **12**[2], 281–318. See also discussion and rejoinder in Fienberg, Haviland, Heckerman, Shachter, Kadane, Moral, Pearl (2003).

— (2004): *Robustness of causal claims*. In: *Uncertainty in artificial intelligence, proceedings*. Ed. by J. Halpern, C. Meek (AUAI Press, Arlington), 446–453. arXiv:1207.4173.

— (2009): *Causality: Models, Reasoning, and Inference*, 2nd ed. (Cambridge University Press, Cambridge). First publ. 2000.

Pearl, J., Mackenzie, D. (2018): *The Book of Why: The New Science of Cause and Effect*. (Basic Books, New York).

Simon, H. A. (1954): *Spurious correlation: a causal interpretation*. J. Am. Stat. Assoc. **49**^{267}, 467–479.

Spiegelhalter, D. J., Grieve, A. P., Lindley, D. V., Lindley, D. V., Copas, J. B., Cairns, A. J. G., Chatfield, C., Box, G., et al. (1995): *Discussion of the paper by Draper [Assessment and propagation of model uncertainty]*. J. Roy. Stat. Soc. B **57**^{1}, 71–97. See Draper (1995). https://classes.soe.ucsc.edu/ams206/Winter05/draper.pdf.

Toda, M. (1977): *Causality, conditional probability and control*. In: Aykaç, Brumat (1979), p. 7, 109–124.

Valdes-Sosa, P. A., Roebroeck, A., Daunizeau, J., Friston, K. (2011): *Effective connectivity: influence, causality and biophysical modeling*. NeuroImage **58**^{2}, 339–361.

Wasserstein, R. L., Lazar, N. A. (2016): *The ASA's statement on p-values: context, process, and purpose*. Am. Stat. **70**^{2}, 129–133. See ASA (2016) and discussion in Greenland, Senn, Rothman, Carlin, Poole, Goodman, Altman, Altman, et al. (2016). https://catalyst.harvard.edu/pdf/biostatsseminar/ASA_s_statement_on_p_values_context_process_and_purpose.pdf.
