# Supplementary material for: Robust point-process Granger causality analysis in presence of exogenous temporal modulations and trial-by-trial variability in spike trains
Source: PLoS Comput Biol. 2021 Jan 25;17(1):e1007675. doi: 10.1371/journal.pcbi.1007675 (PMC7861554; doi:10.1371/journal.pcbi.1007675)
Supplement: Attachment — Submitted filename: ResponseToReviewers.pdf [file pcbi.1007675.s005.pdf]

Reviewer 1

Reviewer 1 performed a very in-depth evaluation of our manuscript providing several advices to improve its quality. In particular, he suggested to perform additional simulations as well as edits to the main text to improve the theoretical framework of our results. We carefully revised our manuscript following his advices. Below, we provide a point-by-point description of how we addressed his comments and suggestions. We use *italic text* to report verbatim his comments.

1. The examples (mostly summarized in Figs 2, 3) demonstrating the model emphasize the reduction of false positives as compared with Kim et al. What about false negatives, especially with weak exogenous input? Some readers will fear that the model may underestimate Granger-causal connections that are 'truly' there. I recommend to include the results for networks like the ones of Figs 2 and 3 but without exogenous input, or with a much weaker one. If the authors have no time to perform such additional simulations, can they at least explain, in the Introduction and Discussion, that the model hasn't been tested for false negatives with weak exogenous inputs?

Reviewer 1 is correct in pointing out that in our manuscript we focused our attention predominantly on the reduction of false negatives of our model compared to Kim et al.'s and we did not investigate the complementary issue of the sensitivity of our method. That is, its capability to detect a functional connection when it is truly there.

To address this point, we focused on two factors that directly modulate G-ETM's sensitivity in detecting an existing functional connection: the strength of the connection and the number of available trials. We then performed a series of Montecarlo simulations of a simple network consisting of two units with a functional connection from neuron 1 to neuron 2. In these simulations, we parametrically changed the values of these two parameters and assessed the performance of our G-ETM method. Results are shown in a new figure (Fig. 6) and discussed in a newly added section ("Sensitivity of G-ETM") on pags. 9-10 of our revised manuscript. They show that G-ETM has as high sensitivity and it can detect a functional connection from a source to a target neuron even when the effects of this connection are too weak to produce any appreciable change of the target neuron's firing rate.

2. The reported percentages for the results of the simulation of Fig. 4 are unclear (lines 136–144). For example, what do the 42% and 4.2% proportions of false positives refer to? are they over the total number of connections x trials, 16 x 100? or over the trials with at least one wrong

connection? Same holds for the 85% and 88%. Analogous questions arise for the simulation described on lines 216–224.

In the revised version of our manuscript we now provide the details of how the percentage of correct responses and false positives were computed. In particular, we added the following sentence on pags. 7-8: "To this end, we computed the percentage of correct responses by dividing the number of correctly detected functional connections by 6 (the ground-truth value of functional connections present in the network) and the percentage of false positive by dividing the number of incorrectly reported functional connections by 10 (the ground-truth value of non-connected node pairs)."

3. It's surprising and interesting that the fitting of the model of eq. (4) doesn't end up setting the γ-terms to zero and using the exogenous α-term only, which offers a large amount of degrees of freedom. Were any regularizers or bounds used in the fitting? Or is this the effect of Akaike's criterion? in the latter case, how large was N_i thus found? It'd be useful if the details of the fitting, only generically mentioned on lines 361–369, were reported in more detail.

4. I don't understand how the model of eq. (6) can be fitted at all. For this model, one set of trials (such as the 40 trials of Fig. 4) constitutes one data point – surely so for the fitting of the A-parameters. In the example of lines 212–222 the model therefore seems to be fitted with one data point From a 'frequentist' viewpoint such fit hardly seems possible. From a Bayesian viewpoint it is possible: the prior information will simply play a dominant role in the fitting. But this prior information should then be explicitly stated. How are we to interpret the fitting procedure in the present case?

Here, Reviewer 1 requests further clarification and raises very interesting points concerning how our models are fitted to the data. To address them, let us first consider that the GLM fitting process needs as inputs an $m \times n$ predictor matrix X and an n-dimensional response vector Y. Each time point of the spike trains contributes a row in the matrix X and n is equal to the total number of to-be-estimated parameters (i.e. the γ, α and β in Eqs. 4 and 6).

In the case of Eq. 4 we used no additional regularizers or bounds and how the "variance" of the spike trains is distributed among the γ and α parameters was a result of the GLM fitting process. Indeed, the γ and α terms in Eq. 4, model two different components of the neuronal conditional intensity function (CIF). To better understand this point, let us first make two considerations. First, Poisson spike trains possess, even in the presence of no exogenous cause, an intrinsic temporal variability which is, in general, not correlated across trials. Second, the γ terms model how much a source neuron's spiking activity before time t, integrated over an appropriate time

scale, can explain a target neuron's firing probability at time t. Thus, the fitting process will assign non-zero values to the γ terms when the source and target neuron's variability in firing rate are related on a trial-by-trial basis (e.g., on a trial-by-trial basis, an increase/decrease in the firing rate of one produces an increase/decrease in the firing rate of the other). The α terms model instead components of the CIF that are (1) consistent across trials and (2) cannot be attributed to interactions with other units, otherwise the fitting process would assign that component to the γ terms as that would lead to a bigger increase of the spike trains' likelihood. Thus, given that they represent different components of the neuronal CIF, the GLM fitting process "distributes" the variance of the spike trains on both the γ- and α-terms, without "assigning" it only to the γ-terms.

The value of the hyper-parameter N_i (i.e. the number of α parameters) depends, in general, on the characteristics of the exogenous modulations. For example, in the case of Fig. 2 in the manuscript, we have $N_1 = N_2 = 15$ (the number of red dots in the two panels of Fig. 2B). In general though, N_i can be different across units and its value is set based on Akaike's information criterion.

Finally, Reviewer 1 is correct in noticing that convergence in the fitting of Eq. 6 is, per se, not ensured. For this reason, in our simulation, we added two regularizers that constrain the GLM fitting process. This implementation detail was not included in our original submission. Following his suggestion, we added it in the revised version of our manuscript. The relevant text is on pag. 18 of our revised manuscript.

5. Important question: why the linear dependence of the two models, eqs (4) and (6)? What happens with different functional forms? How uncertain are our conclusions about causal links, when we consider our uncertainty about the structure of the model? The Monte Carlo simulations of lines 136–144 and 212–222 were generated by a process having essentially the same form as the model, as explained on lines 387–399. So they 'validate' the model only in the sense of ascertaining the absence of bugs in its numerical implementation. They don't validate the model in the sense of its appropriateness for real data. No such validation can in fact exist: it would require knowledge of the real-data 'ground truth' – and if we knew the ground truth we wouldn't be here making models. Such validations, in this sense, are always circular; their purpose is only to give a pat on the back. It is known (Draper 1995; Chatfield 1995) that the uncertainty in the model itself (also called structural uncertainty) is 'likely to be more serious than other sources of uncertainty' (Chatfield), 'leading to inaccurate scientific summaries and overconfident decisions that do not incorporate sufficient hedging against uncertainty' (Draper). It should preferably be assessed –

and this is indeed possible, see the cited papers – or at the very least pointed out and discussed. What are the results of the models on data generated by a different, synthetic but more realistic model, say a leak-integrate-and-fire one?

In his first question, we assume that Reviewer 1 refers to the linear dependence of our model from the history parameters (the R terms in Eqs. 4 and 6)[1]. The reason for this choice, is because the estimation of neuronal conditional intensity functions has been historically cast in a linear autoregressive framework [1] as the convolutions of neuronal activities with a linear kernel. In general, however, the dependence of our model from the R-terms can have any functional form.

In the subsequent part of his comment, Reviewer 1 raises many very valid points concerning the robustness of our models that are, as he also acknowledges, inherent, in some way or another, to any model. We addressed them in two ways.

First, we performed additional simulations in which spike trains were generated by means of a network of integrate-and-fire units. In this network, the spike generation process departs in significant ways from the assumptions of our models, as discussed on pags. 8 and 9 of our revised manuscript. Yet, results in a newly added figure (Fig. 5), show that our model can detect its functional connectivity with both high accuracy and a percentage of false positives below the set statistical threshold. These new simulations show that G-ETM is robust with respect to violations of its assumptions.

Second, we added text that discusses, along the lines suggested by the Reviewer, the more general issue of uncertainty about the structure of a model. The relevant text is in the Discussion section (pag. 16) of our revised manuscript.

6. I appreciate the mathematical explanations (e.g. around lines 78 and 95), in terms of number of parameters, of why Kim et al.'s method can infer misleading Granger-causal links, whereas the proposed methods can avoid this. But the deeper and more general reason – connected with the very notion of 'causality' – surprisingly isn't mentioned. And this reason is important to understand what we're doing with our modelling. Phenomena or situations B1, B2, . . . are considered the causes of event A if, roughly speaking, (i) all Bi precede A in time, (ii) knowledge of the Bi changes our uncertainty about A, (iii) knowledge of other phenomena C1, C2, . . . preceding A, besides the Bi, doesn't change our uncertainty about A. The

[1] The linear dependence of our model from the α, β and γ parameters is a necessary condition to apply generalized linear models.

last two conditions are expressed as:

$$P(A|I) \neq P(A|B1, B2, \ldots, I) = P(A|B1, B2, ...; C1, C2, ..., I)\ \forall\ Ci \quad (1)$$

where I represents contextual knowledge necessary to define the problem. Such a generic definition appears to be more or less agreed upon by many studies, from Freedman's (1991; 1997; 2004; 2006) to Pearl's (2009;2003; 2004; 2018), from Simon's (1954), Good's (1961a,b), or Toda's (1977) to Valdes-Sosa et al.'s (2011). A very interesting perspective is given by Lindley and Novick (1981). The invariance represented by the second equality expresses the 'robustness' of causal links, when compared with non-causal correlations. Hence the testing of such robustness by interventions, as opposed to observations (Pearl & Mackenzie 2018 ch. 3, explain this very tersely in my opinion – even if one doesn't wholly agree with their whole work). The numerical values of these conditional probabilities can be compared in various ways (means, variance, tests, etc.).

In Granger's (1969 § 5) binary study, B1 is the history of previous events of the same kind as A, the term C1 is the history of a different kind of events, and others B2, C2, . . . are assumed not to exist. So he can simply compare the conditional probabilities P(A — B1, I) and P(A — B1; C1, I) above (ibid. p. 429) to ascertain whether C1 is or isn't a cause of A, besides B1. If we know that other phenomena B2 or C2, such as extra-network inputs in a neural network, are present, we can't use Granger's binary case; that'd be a misapplication. The point is not that it can give 'wrong' results. The point is that whatever results it gives are meaningless, because the conditions for the definition and quantification of causal links are not met. For this reason Kim et al.'s method would be misapplied if used for a non-isolated neural network, and one needs the method proposed in the present manuscript (putting aside problems with model uncertainty, point 5 above).

We agree with Reviewer 1 that the results of Fig. 1 need a more theoretically-grounded discussion. To this end, we added a paragraph on pag. 5 of our revised manuscript where we state, along the lines suggested by the Reviewer, that :

"At a theoretical level, in the Granger framework, events B_i are said to be the "cause" of another event A if (1) they precede A in time, (2) knowledge of the B_i changes our uncertainty about A beyond inclusion of all other available information C_i. This can be expressed in mathematical notation in terms of conditional probabilities as: $P(A) \neq P(A|B_1, B_2...) = P(A|B_1, B_2..., C_1, C_2, ...)$, where $P(A)$ is a probability distribution representing our knowledge of the event A and $P(A|\mathbb{D})$ is the probability of A conditioned on a set $\mathbb{D}$ of events. If, however, relevant events are not included in the set B_i then this might lead to spurious causality links [2] as the union of the events B_i and C_i no longer represents all available in-

formation, as assumed by the Granger framework. In Kim et al.'s Granger causality method, the exogenous modulations of neuronal spike trains are not included in the predictor set B_i [3] and its application to such data sets becomes thus meaningless."

7. Generalized linear models constitute an infinite-dimensional family of parametric models. It's extremely vague to say that we're using a generalized linear model. Laplace (1809—1810) could have said so too, since he used normal distributions. Saying so could be informative when we imply the existence and role of some sufficient statistics. But no such connection is made in this manuscript. I'd appreciate if at least the link function were specified when mentioning a general linear model.

We acknowledge that, in the previous version of our manuscript, important details about the used GLM models were missing and we apologize for that. On pag. 17 we now specify: "Model parameters are estimated by means of a GLM fitting process with a binomial distribution function and a logit link function".

8. How is 'information flow' (e.g. line 27) defined? what is it exactly?

In our original submission we incorrectly used "information flow" as a synonym of "functional connectivity". We have however realized that this might be confusing for the reader and thus, in our revised manuscript, we now only use the expression "functional connectivity".

9. Why is the difference of 85% vs 88% of G-ETM vs Kim (line 142) not significant, but the similar difference of 97% vs 92% of G-ETM vs G-ETMV is?

This comment from Reviewer 1 made us realize that, while data were correctly plotted in Fig. 4, they were incorrectly reported in the main text. The percentage of correct responses for both G-ETM and Kim et al.'s methods is approximately 94% (and not 85% vs 88% as incorrectly reported in the previous version of our manuscript), which explains the difference in significance noted by the Reviewer.

In this revised version of our manuscript, the text now correctly describes the results of Fig. 4B. We apologize for any confusion that our error might have generated.

10. Figure 3 E, plot in 2nd row, 1st column: the blue curve (estimated interaction) seems to extend outside the plot range. How much? Can the plot range be fixed, to include the whole blue and black curves?

We apologize for this glitch in Fig. 3. We changed the vertical range of the plots to include all data points.

11. Figure 7 B, scatter-plot: the points are so many that they overlap, masking their high-density region. I recommend subsampling them so that readers can compare the red line and the density peak in the scatter-plot.

Following Reviewer 1's advice, we replotted Fig. 7B subsampling, by a factor 3, the number of dots.

12. All plots are extremely small. Many readers print articles to read them; they'd be thankful to have larger, decipherable plots.

To address this comment we changed, the layouts of Figs. 3 and 7 (Fig. 5 in the previous version of our manuscript) so as to magnify them along the horizontal axis. That was not unfortunately possible for Fig. 8 (Fig. 6 in the previous version of our manuscript) and the newly added Fig. 10. For the reader's convenience, we thus provide a rotated and magnified version of them as supplementary information (Figs. S2 and S3 respectively).

13. Please see the recent official statements by the American Statistical Association (asa 2016; 2019):

It is time to stop using the term "statistically significant" entirely. Nor should variants such as "significantly different," "$p < 0.05$," and "non-significant" survive, whether expressed in words, by asterisks in a table, or in some other way. [. . .] Where p-values are used, they should be reported as continuous quantities (e.g., $p = 0.08$). (asa 2019 § 2)

I therefore recommend that the authors explicitly state the p-values they find, rather than reporting 'significant' vs 'not-significant'.

Reviewer 1 is absolutely correct. We used terms such as "significant" or "non significant" because they are still common practice in the neuroscientific literature. However, following his comment, in our revised manuscript we now simply report the p-values without further commenting on their "significance".

14. I genuinely thank the authors for limiting the number of acronyms. It was pleasant to read the manuscript without having to continuously jump back and forth trying to find where an unmemorizable random string of letters was defined. I recommend that the two models' acronyms be spelled out directly on lines 38–40.

The manuscript is well written and reads smoothly. It would be practical if eq. (4) were somehow written in full before line 101, where the

γ-parameters are mentioned, or at least if these parameters were explained somewhat more around that line.

On line 27: 'the brain state is perturbed by an event' – can we really speak of 'perturbations', as if it was a stationary state? I think that the message conveyed around that line is valid even without speaking of "perturbations".

There are commas placed in strange, ungrammatical positions here and there; for example: 'the brain state, is perturbed' (line 27),
'because, the GLM' (line 77),
4'This means, that' (line 123),
'this happened because, trial-by-trial' (line 191).
Line 48: it took me some time to understand that 'endogenous, temporal' weren't adjectives of 'modulations'. I'd recommend changing this sentence to 'we will denote temporal modulations [. . .] as endogenous, and temporal modulations [. . .] as exogenous'.

We thank Reviewer 1 for his appreciation of our paper. We addressed all the points that he raises here. In particular:

- We added a table (Table 1) at the beginning of the Results sections showing our G-ETM and G-ETMV models vis-a-vis Kim et al.'s original method. In this manner, Eq. 4 is now introduced before the γ parameters are discussed in the text.

- We corrected the text on line 27, which now states "... the brain changes its state as a consequence of, for example, sensory stimulation or motor behavior"

- We corrected the misplaced commas and we streamlined the sentence highlighted by the Reviewer, which now reads: "Throughout this section we will denote temporal modulations in neuronal responses that are due to interactions between the recorded neurons (including self-interactions) as *endogenous* and temporal modulations that are due to unobserved causes as *exogenous*.".

Reviewer 2

Reviewer 2 requested several clarifications concerning our model and suggested edits to the main text. She/He also requested to perform additional simulations to better characterize the robustness and sensitivity of our proposed point-process Granger causality techniques. We carefully revised our paper following her/his advices. Below, we provide a point-by-point description of how we addressed her/his comments and suggestions. We used *italic text* to report verbatim her/his original comments.

- *The writeup for the results highlight that considering the effect of exogenous events in each non-overlapping window during each trial predicts the functional connectivity between neural units. However, none of the equations referred to (not even the ones proposed) appear in the main text, rather they all are provided under Methods. As a reader this leads to confusion: how to interpret the proposed model from the text without visualizing the actual mathematical change proposed to the existing technique.*

- *Building on the above: it would be *very* useful to have some sort of schematic showing a clear comparison between the proposed and prior modeling frameworks. Seeing the core equations side by side, e.g., in a Table, would allow the reader to clearly delineate the mathematical changes and understand the conceptual contribution of the proposed method.*

We apologize if, in the previous version of our manuscript, the details of our proposed Granger-causality methods were not appropriately described in the Results section. To address this point, in our revised manuscript, we added a table (Table 1) at the beginning of the Results section where we show the formulas of the Granger causality model originally proposed by Kim et al. and our two novel G-ETM and G-ETMV methods. We also added text at several points on pags. 3-6 of the Results section to introduce these formulas.

- *Figure 2 includes caption for subfigure C, i.e., estimates of the exogenous components of firing patterns, the subfigure itself is not shown in the current version.*

In the caption of Fig. 2 the text describing panel B was incorrectly labeled as referring to panel C, while the sentences describing panel B, i.e. those referring to the interaction functions, were included by mistake. Indeed, when no connection is detected by our Granger causality method, as is the case of the results shown in Fig. 2, the estimates of the exogenous components have no meaning and must not be considered.

We apologize for any confusion that our mistake has potentially generated. In the revised version of our manuscript, we corrected the caption

of Fig. 2, which now describes the ground-truth and estimated connectivity patterns (panel A) and the exogenous components estimated by our G-ETM method (panel B).

• *The authors claim that the simulations leading to Figure 3 depict the scenario when simultaneous recording from two different areas is done during an experiment. The idea is to investigate the nature of functional connectivity between subpopulations during such experimental setup. The ground truth model however did not have any interpopulation interactions and what the simulation suggests is that the introduced method could identify that there were no connections between subpopulations. However, it fails to capture how this method(G-ETM) would perform if there were weak/strong interactions between the two subpopulations. Intuitively this should be explored as well. Alternatively, if there are any reasons for omitting such connectivity in model simulations that should be stated.*

Here, Reviewer 2 raises a very important point related to the sensitivity of our G-ETM method. That is, she/he is asking how likely is G-ETM to detect a functional connection known to exist between two units. To address this point, we performed additional simulations in which we parametrically investigated G-ETM's performance in detecting a functional connection existing between two units as a function of two relevant parameters: the strength of the connection and the number of available trials.

We report the results of these simulations in a new figure (Fig. 6) and section ("Sensitivity of G-ETM") on pags. 9-10 of our revised manuscript. In brief, our additional simulations showed that our G-ETM method is sensitive enough to detect a functional connection between two units even when this is too weak to cause any significant change in the target neuron's firing rate.

• *The point about self and mutual interactions being inhibitory/excitatory over different timescales needs further clarification/explanation.*

Following Reviewer 2's suggestion we now provide a more thorough description of the results of Fig. 5 (Fig. 7 in our revised manuscript). In particular, we now state: "This temporal dynamics is compatible with a scenario in which source neurons cause an increase in firing probability of target neurons (late excitatory component) after an initial transmission delay of their influence (early inhibitory component). The extent of these delays can be estimated based on the time at which the curves in Fig. 7C cross the x-axis."

• *The discussion section does not include any discussion about the limitations/ potential sources of error while using these models that is if there*

are certain situations when the functional connectivity between neural units are not faithfully recovered using this method. In particular, the proposed method chooses a specific functional form for the new exogenous covariates. Are there situations where these parametric forms are expected to fail? Simulating spike trains from a mismatched model, perhaps even a spiking neuronal network model where a variety of modulating factors could be investigated, could be quite useful in this regard.

We addressed Reviewer 2's request of a more in-depth discussion of the potential limitations of our model in two ways.

First, we performed additional simulations in which spike trains were generated by means of a network of integrate-and-fire units. In this network, the spike generation process departs in significant ways from the assumptions of our models, as discussed on pags. 8 and 9 of the revised manuscript. Yet, results in a newly added figure (Fig. 5), show that our model can detect its functional connectivity with both high accuracy and a percentage of false positives below the set statistical threshold, supporting its robustness with respect to violations of its assumptions.

Second, we added a paragraph and a figure (Fig. 10) to the Discussion section. Therein, we describe cases in which G-ETM and G-ETMV either cannot be applied (First and second point of the newly added paragraph) or are most likely to produce incorrect results (third point and Fig. 10). The relevant text and figure are on pag. 15 of our revised manuscript.

- *In the Abstract/Intro there is some confusion regarding what is meant by 'exogenous'. Spike rate modulation may not actually be exogenous to the population; rather it is exogenous to the prior granger formulation. Would it be better to simply refer to these as unmodeled factors?*

We see Reviewer 2's point here and we agree with her/him that using the term "unmodeled factors" is more appropriate in the Abstract and Introduction sections. That said, we refer to firing rate modulations throughout our paper. While it would be more appropriate to refer to them as "unmodeled" in the Abstract and Introduction sections, where our novel Granger causality methods have not been fully described yet, the same would not apply to subsequent sections of our manuscript in which those factors are actually included in our model. Therefore, for reasons of consistency, we kindly ask the Reviewer to let us maintain the current terminology.

- *The Introduction could use a little more in terms of background on prior Granger methods. The authors introduce the abbreviations for their proposed techniques without actually defining them; it would be useful to get some initial context on what these methods are going to do (e.g., by adding new covariates to the point process framework).*

Following Reviewer 2's suggestion, in the Introduction section, we now provide both the definition as well as a brief description of both G-ETM and G-ETMV. The relevant text is on pag. 3 of our revised manuscript.

- *The notation is Equation (4) is somewhat nonstandard. I think this would be easier to follow if c was defined as an integer and used in the equation, with the $(t/T)N_i$ definition being introduced later. Why are some parameters notated with uppercase while others lowercase?*

We agree with Reviewer 2 that our notation in Eq. 4 was potentially confusing and we modified both Eqs. 4 and 6 following her/his suggestion. In addition, from her/his comment, we also realized an inconsistency in the way we used uppercase and lowercase variable names. It was our intention to use an uppercase notation for variables that are directly computed from the data (e.g. the R terms in Eqs .2, 4 and 6) and a lowercase notation for variables that are instead estimated by the GLM fitting process (e.g. the α terms im Eqs.4 and 6). This notation was however broken in Eq. 6. We have thus modified Eqs. 4, 5, 6, and 7 to follow the above notation. We also added a sentence at the beginning of the Materials and methods section (pag. 16) of our revised manuscript that explicitly states the meaning of uppercase and lowercase variable names.

References

[1] Okatan M, Wilson MA, Brown EN. Analyzing functional connectivity using a network likelihood model of ensemble neural spiking activity. Neural Computation. 2005;17:1927–1961. doi:10.1162/0899766054322973.

[2] Granger CWJ. Investigating causal relations by econometric models and cross-spectral methods. Econometrica. 1969;37(3):424–438.

[3] Kim S, Putrino D, Ghosh S, Brown EN. A Granger causality measure for point process models of ensemble neural spiking activity. PLoS Computational Biology. 2011;7(3):e1001110. doi:10.1371/journal.pcbi.1001110.
