# Supplementary material for: Robust point-process Granger causality analysis in presence of exogenous temporal modulations and trial-by-trial variability in spike trains
Source: PLoS Comput Biol. 2021 Jan 25;17(1):e1007675. doi: 10.1371/journal.pcbi.1007675 (PMC7861554; doi:10.1371/journal.pcbi.1007675)
Supplement: Attachment — Submitted filename: ploscb-reviewer1_round2.pdf [file pcbi.1007675.s006.pdf]

Review of PCOMPBIOL-D-20-00115 – version 2

I thank the authors for their patience and the additional work they did with the point-neuron simulations, the new explanations, and the initial table with the model equations.

On my part I only recommend the addition of a couple of clarifications, discussed in points 1–3 below. Points 1 and 2 are only recommendations, not requirements. Point 3 reports some errors, so I would ask the authors to fix at least those.

But I want to add that the authors are, in my opinion, pushing their approach by emphasizing wrong or questionable reasons and "tests", while sadly de-emphasizing the good and interesting parts. I say this in good faith, because I find the approach and model useful. My concerns are discussed in points A1–A3. These are *not* requests for modification. It is up to the authors to decide if they find my concerns reasonable and if they want to address them in this work, or maybe in future works, or just disregard them.

1 Functional or anatomic connectivity?

The authors stress from the beginning the use of the method to infer *functional* connectivity. This notion – a sort of time-directed statistical correlation, defined in vague and partly contradictory ways in the literature (e.g. Valdes-Sosa et al. 2011; Stokes & Purdon 2017; Faes et al. 2017; Barnett et al. 2018) – stands in contrast with *anatomical* connectivity. Neurons can be functionally connected even if they are not anatomically connected, and vice versa. But the present authors seem to conflate the two kinds of connectivity as the manuscript progresses, for example in the application of Fig. 5. Also, some authors (e.g. Granger 1988 himself; Demirci et al. 2009) seem to consider functional connectivity as a continuous, rather than binary, notion.

I think that readers would appreciate if the present authors made their stance about functional connectivity and Granger causality more precise. The clarity of their stance is important because it determines tests, evaluations, and applications of their method. For example, was the application to integrate-and-fire units, Fig. 5, judged based on the physical connections of the units? if so, why cannot the functional and physical connections of the units be different? (see point A3 below.) Do the green-black diagrams present in many figures imply that the authors see functional connectivity as a binary quantity? And how can a 'ground truth' *functional* connectivity be experimentally established? See point A3.

2 Stationarity

In the Discussion and around lines 475ff. the authors refer to non-stationarity as the reason why an extension of Kim et al.'s model is needed. I do not think that stationarity is an issue for any of the models (2), (4), or (6). As I see it, time-stationarity is needed for a well-defined frequency analysis, and therefore only necessary for frequency-based methods (such as Granger's initial one, Geweke, and others), not for the methods of the present manuscript. – But I have not carefully analysed this mathematically, so I can be wrong.

Regarding the statement on line 367: "The jointly stationarity assumption gives Granger causality several appealing characteristics [ref. to Seth et al.]", I believe the authors are referring to Seth et al.'s statement "Given these assumptions, G-causality has several attractive properties". Note, however, that the *given* in that statement does not mean *because of*, but *in the context of* – or even *notwithstanding*. So their meaning is almost opposite.

The real reason why model (2) needs to be extended is because it does not include quantities that we know are potential causes. That is, it violates the basic Granger-causality assumption that

$$\mathrm{P}(A \mid \bar{B}) = \mathrm{P}(A \mid \bar{B}, \bar{C}, \bar{D}, \dots) \quad \text{for any other quantities } \bar{C}, \bar{D}, \dots \tag{r1}$$

where $\bar{B}$, $\bar{C}$, $\bar{D}$ denote disjoint sets of time-dependent quantities. See the discussion in Granger (1988), especially p. 200, and also Granger & Thomson (1987). Model (2) assumes that no other input quantities besides the activities of the units can influence the system; that is,

$$\bar{B} \coloneqq \text{activities of the units}, \quad \bar{C} \coloneqq \text{external inputs}, \quad \bar{D} \coloneqq \text{anything else},$$
$$\mathrm{P}(A \mid \bar{B}) = \mathrm{P}(A \mid \bar{B}, \bar{C}, \bar{D}) \tag{r2}$$

the last equality being clear from the fact that model (2) does not have any parameters besides those related to the units (the term $\gamma_{i,0}$ makes the self-influence of unit i affine instead of linear). But we know that units are influenced by external units and stimuli:

$$\bar{B} \coloneqq \text{activities of the units}, \quad \bar{C} \coloneqq \text{external inputs}, \quad \bar{D} \coloneqq \text{anything else},$$
$$\mathrm{P}(A \mid \bar{B}) \overset{!}{\neq} \mathrm{P}(A \mid \bar{B}, \bar{C}) = \mathrm{P}(A \mid \bar{B}, \bar{C}, \bar{D}, \dots) \tag{r3}$$

so that requirement (r1) is violated by $\bar{C}$. To satisfy it again we must include $\bar{C}$ with $\bar{B}$. Model (4) does this by adding the time-dependent terms α.

In passing I may add that such inclusion could also have been done by adding a real-valued term $R_{0,m}(t)$ representing external input. Such a term would have been externally given if known (this is essentially what is done in Mimica et al. 2018 although they do not care about Granger causality), or inferred in block with the corresponding $\gamma_{i,0,m}$ if unknown; model (4) does the latter.

In this regard, the new statement around lines 96–100 misses the point: It is not that "the union of the events $\bar{B}$ and $\bar{C}$ no longer represents all available information", but that $\bar{C}$ turns out to be relevant, eq. (r3), whereas before it was assumed to be irrelevant, eq. (r2). See again the initial discussion in Granger (1988).

3 Additional points

- I recommend that the application to integrate-and-fire units of lines 182ff. be mentioned and even emphasized in the Abstract.

Please note that many works in the literature (Averbeck 2009; the book Grün & Rotter 2010 and refs therein; Brette 2015 fig. 2A) report that real neurons in many circumstances or brain areas are *not* Poissonian, so some readers will disagree with the justification for the modifications mentioned on lines 561–566. It is a pity that results are not reported for the case without modifications; they would have been extremely interesting, no matter the results. But I am *not* requiring the authors to report them. See also point A2 below.

- In Fig. 4 and line 157, please make clear according to which distribution the "random" generation occurs.

- $A_{i,p}$ on lines 313–325 should be β?

- Please note that "Montecarlo" is spelled 'Monte Carlo' (according to Oxford, Cambridge, Merriam-Webster). There are some strange commas across the paper, e.g. "If," line 95, "emphasized, that" line 244.

* * *

Here follow my personal concerns. They are not meant as recommendations for amendment. But I share them with the authors in case they might find them useful.

A1 "Validation"

A parametric model such as eq. (2), (4), or (6) is a manifold of probability distributions within a larger space, more precisely a simplex (in this case a simplex of 'point processes'). By definition, a long run of samples from a specific distribution chosen from the model defines a unique point in that manifold. And a fitting/optimization algorithm must obviously recover that unique point in that manifold.

A model such as eq. (2) is a proper submanifold within the manifold of the more general model eq. (4). They are nested models. A fitting algorithm for the submodel can therefore never recover a point generated by a distribution chosen from the more general model, unless that distribution is itself an instance of the submodel.

These are mathematical tautologies.

Numerical implementations of the random generator or of the fitting procedure can of course go wrong: they may have bugs or converge too slowly for practical purposes. The mathematical tautologies above – which *must* be satisfied by the numerical implementation – are therefore used to check for bugs and convergence.

Figures 1, 2, 3, 4, 8 and related discussion in the manuscript validate that the *numerical algorithms* used by the authors satisfy these basic requirements. Therefore, if these checks failed they would invalidate the optimization algorithm (or the random generator), *they would not invalidate the model*. So the statements on line 178 and line 326, "the model is validated. . . ", are incorrect. Any model perfectly fits itself. Points deliberately chosen in the manifold are obviously in the manifold.

Now if the purpose of the authors is to make their numerical optimization code publicly available for use, then it is important that they show that their code is bug-free and converges, and those figures and their discussion are justly emphasized.

But this mathematically guaranteed convergence tells us nothing whatsoever on how a model fares with real data, that is, with points outside the model's manifold.

So, if the purpose of the authors is not to share a new numerical optimization algorithm, but to propose the use of the models (4) & (6), then Figs 1, 2, 3, 4, 8 and their discussion are somewhat superfluous. They could not have been otherwise, no matter the model. Of course the readers expect the numerics to be correct; it is nice that the authors show that they are correct, but this could have been done in supplementary material.

In this case a reader will wonder "why are the authors emphasizing this tautology?". Some readers may suspect that the authors are trying to deceived them; other readers may get the impression that the authors do not have a full statistical grasp of what they are doing. These possibilities would be detrimental to the model and to the manuscript.

(It's as if you wanted to convince me that you're really good at finding lost keys, and as a proof you put a key in your pocket and then tell me "Now I'll find the key", and take it out of your pocket. Well, no surprise, you put the key there yourself! Are you making fun of me? This doesn't tell me anything about your skills in finding lost keys.)

Again, I think that the proposed approach and model *are* useful. Therefore it would be sad if any readers of this manuscript felt such kind of misgivings.

A2 Robustness and applications

The interesting and non-tautological part, instead, is the application to real spike-train data (Fig. 7). The interpretation and discussion of the results, around lines 275–289, is also very interesting. It is a pity that it is left to so late in the manuscript.

The statement "Although in this case we do no have the ground-truth connectivity pattern, quantitative simulations reported in Figs. 3 and 4 strongly suggest that these additional causal connections are likely false positives" is just wishful thinking, however. For two reasons. First, because we are speaking about *functional*, not anatomical, connectivity. Is there any "ground truth" for it? See points 1 and A3. Second, because the simulations of Figs 3 & 4 were generated by the general model itself: as discussed in point A1 they don't prove anything about model reliability or generalizability.

What is interesting, instead, are the conditional inferences that we can make by comparing the two models: (I) *If* there are no exogenous influences, then the sub-model (2) – which would be the most appropriate in this case – indicates some specific internal functional influences, for example from source 6 to target 10. (II) *If* there are exogenous influences, then the general model (4) – which would be the most appropriate in this case – indicates that no such functional influences occur. In my opinion this is an interesting inference, even if it has a conditional character. In the present case we can consider case (I) to be unlikely (but not impossible) for neurobiological reasons.

Also very interesting is the new application to the artificial network of integrate-and-fire neurons. It would have been interesting to see the inferred functional influences also in this case, similarly to Fig. 7A–B. I am not sure whether Fig. 5 provides any interesting information: again the issue is the difference between anatomical and functional connectivity; see point A3 below.

As I said before, for these simulations it is a pity that the authors made the (unrealistic) modifications described on lines 561–566. It would have been interesting to see the results with and without the modifications.

Finally, also the application of Fig. 10 is extremely interesting. What's funny is that the authors interpret it as an erroneous conclusion on the model's part, whereas I actually suspect that the model did not perform so badly after all. My feeling is that a full (Bayesian) probabilistic analysis would reveal that the model was, so to speak, "undecided" in assigning the correlations to exogenous or to endogenous influences, and that the latter prevailed only by little. This "indecision" could appear as a bimodality in the posterior distribution of the model's parameters, with the second mode (endogenous interpretation) only slightly higher than the first (exogenous interpretation). That is, the curves of Fig. 10C–D could have large error bars around them, actually representing a bimodal distribution in the y-direction. The second mode would be centred on values with small interactions and smaller exogenous components.

This would be a very interesting result and a case *in favour of*, not against, the model. Unfortunately p-values and significance tests typically miss such important inferences (which is why the American Statistical Association is discouraging their use).

Obviously the measures of "influence" mentioned so far are conditional on the assumptions of the model, but such kinds of assumptions are almost always inevitable. This is related to point A3 below.

A3 Functional vs anatomical connectivity and meaning of the model

As mentioned in point 1, functional and anatomical connectivity are distinct notions. This distinction is important for the evaluation of Granger-causality models. And it has important consequences for the judgement of the model proposed in the manuscript.

'Functional connectivity' is not a well-defined notion. Just like 'interaction' or 'uncertainty' it can be defined and measured in different ways, all possibly useful in different applications.

My point of view is that the autoregressive model discussed in the manuscript provides one way to *define* and *measure* functional connectivity. This remark is valid for any model used for Granger causality. The original studies, for example, focused on what could be called "linear" Granger causality. The present model measures functional connectivity based on a specific autoregressive picture of a neuron's firing rate. Several models may be useful and comparing their measures is useful too. And none of such models can be, or can be meant to be, a physical model: neurons work in completely different ways, dictated by the laws of electromagnetism and chemistry (see Diaconis et al. 2007 for a brilliant discussion about a similar point).

This is why – in my opinion – the manuscript is focusing too much on unimportant matters such as "validation" – which is *not* provided by self-applications of the model, and even impossible if a ground truth for functional connectivity is undefined – while leaving almost out the really important and useful parts. The point, for me, is not whether the model gives black-or-white answers that coincide with anatomical properties. The model is useful because it *quantifies*, from a specific point of view, time-directed correlations. The specific curves of Fig. 7C–D, with their numerical values, are important. I personally do not see functional connectivity or interaction as a 0-or-1 quantity, so to me the green-black diagrams of Fig. 7A are uninteresting. But of course they are interesting for readers with a binary conception of functional connectivity. For this reason I was also interested in the application of Fig. 5 but without the modifications listed on lines 561–566. (In the case of only two units the two kinds of connectivity must perhaps be equated.)

References

Averbeck, B. B. (2009): *Poisson or not Poisson: differences in spike train statistics between Parietal Cortical Areas*. Neuron **62**^{3}, 310–311.

Barnett, L., Barrett, A. B., Seth, A. K. (2018): *Misunderstandings regarding the application of Granger causality in neuroscience*. Proc. Natl. Acad. Sci. (USA) **115**^{29}, E6676–E6677. See Stokes, Purdon (2018a).

Brette, R. (2015): *Philosophy of the spike: rate-based vs. spike-based theories of the brain*. Front. Syst. Neurosci. **9**, 151.

Demirci, O., Stevens, M. C., Andreasen, N. C., Michael, A., Liu, J., White, T., Pearlson, G. D., Clark, V. P., et al. (2009): *Investigation of relationships between fMRI brain networks in the spectral domain using ICA and Granger causality reveals distinct differences between schizophrenia patients and healthy controls*. NeuroImage **46**^{2}, 419–431.

Diaconis, P., Holmes, S., Montgomery, R. (2007): *Dynamical bias in the coin toss*. SIAM Rev. **49**^{2}, 211–235. http://statweb.stanford.edu/~susan/papers/headswithJ.pdf.

Faes, L., Stramaglia, S., Marinazzo, D. (2017): *On the interpretability and computational reliability of frequency-domain Granger causality*. F1000Research **6**, 1710. See Stokes, Purdon (2017).

Granger, C. W. J. (1988): *Some recent development in a concept of causality*. J. Econometrics **39**$^{1–2}$, 199–211.

Granger, C. W. J., Thomson, P. J. (1987): *Predictive consequences of using conditioning or causal variables*. Economet. Theory **3**^{1}, 150–152.

Grün, S., Rotter, S., eds. (2010): *Analysis of Parallel Spike Trains*. (Springer, New York).

Mimica, B., Dunn, B. A., Tombaz, T., Bojja, V. P. T. N. C. S., Whitlock, J. R. (2018): *Efficient cortical coding of 3D posture in freely behaving rats*. Science **362**^{6414}, 584–589.

Stokes, P. A., Purdon, P. L. (2017): *A study of problems encountered in Granger causality analysis from a neuroscience perspective*. Proc. Natl. Acad. Sci. (USA) **114**^{34}, E7063–E7072. See also Faes, Stramaglia, Marinazzo (2017), Barnett, Barrett, Seth (2018), and Stokes, Purdon (2018a) and correction in Stokes, Purdon (2018b).

— (2018a): *Reply to Barnett et al.: regarding interpretation of Granger causality analyses*. Proc. Natl. Acad. Sci. (USA) **115**^{29}, E6678–E6679. See Stokes, Purdon (2017) and Barnett, Barrett, Seth (2018).

— (2018b): *Correction for Stokes and Purdon, A study of problems encountered in Granger causality analysis from a neuroscience perspective*. Proc. Natl. Acad. Sci. (USA) **115**^{29}, E6964. See Stokes, Purdon (2017).

Valdes-Sosa, P. A., Roebroeck, A., Daunizeau, J., Friston, K. (2011): *Effective connectivity: influence, causality and biophysical modeling*. NeuroImage **58**^{2}, 339–361.
