# Supplementary material for: Robust point-process Granger causality analysis in presence of exogenous temporal modulations and trial-by-trial variability in spike trains
Source: PLoS Comput Biol. 2021 Jan 25;17(1):e1007675. doi: 10.1371/journal.pcbi.1007675 (PMC7861554; doi:10.1371/journal.pcbi.1007675)
Supplement: Attachment — Submitted filename: ResponseToReviewers.pdf [file pcbi.1007675.s007.pdf]

Reviewer 1

Reviewer 1 provided several suggestions on how to improve our manuscript as well as additional theoretical considerations. We use *italic font* to report verbatim the Reviewer's text.

1 *Functional or anatomic connectivity?*

The authors stress from the beginning the use of the method to infer functional connectivity. This notion – a sort of time-directed statistical correlation, defined in vague and partly contradictory ways in the literature (e.g. Valdes-Sosa et al. 2011; Stokes & Purdon 2017; Faes et al. 2017; Barnett et al. 2018) – stands in contrast with anatomical connectivity. Neurons can be functionally connected even if they are not anatomically connected, and vice versa. But the present authors seem to conflate the two kinds of connectivity as the manuscript progresses, for example in the application of Fig. 5. Also, some authors (e.g. Granger 1988 himself; Demirci et al. 2009) seem to consider functional connectivity as a continuous, rather than binary, notion.

I think that readers would appreciate if the present authors made their stance about functional connectivity and Granger causality more precise. The clarity of their stance is important because it determines tests, evaluations, and applications of their method. For example, was the application to integrate-and-fire units, Fig. 5, judged based on the physical connections of the units? if so, why cannot the functional and physical connections of the units be different? (see point A3 below.) Do the green-black diagrams present in many figures imply that the authors see functional connectivity as a binary quantity? And how can a 'ground truth' functional connectivity be experimentally established?

We agree with Reviewer 1 that anatomical and functional connectivity are distinct notions, which should not be conflated. One can have one without the other and viceversa.

Reviewer 1 is correct in noticing that the data sets used to compute results in Fig. 5 were generated by having the spikes of one unit influencing the membrane potential of the other. In doing so, it might seem that we are conflating anatomical and functional connectivity. That is not, however, the case. While real neurons do exchange/process/store information by modulating each other's membrane potentials, the influence that a single real neuron exerts on another neuron is, in general, so small (but see, for example, [1] for a specific system in which this does not hold) that it can be removed without noticeably affecting the network dynamics. That is not the case in our simple network of simulated integrate-and-fire units, where the influence between the two modeled neurons is orders of magnitude stronger than that existing between real neurons. This influence must be thus considered to model functional interactions between two neurons

without any assumption on whether they are physically connected or not. To make this point clearer, we revised the text describing the results of Fig.5. The relevant text is on pag. 8 of the Results section.

At the end of this first section of comments, Reviewer 1 asks two very interesting questions. First, He asks whether we consider functional connectivity to be a binary quantity. This is a highly non-trivial question and addressing it requires an operational definition of how one defines/measures/estimates functional connectivity. In the Granger framework, functional connectivity is estimated by comparing the residual variance produced when fitting a full and reduced model respectively to the data. This comparison yields, in general, a real value (for example, in our study it is the difference of the deviances produced by a full and reduced model respectively). That is, at its core Granger causality is intrinsically not binary. It all depends on how one interprets its results. In a frequentist approach, as that used in our study, this model comparison yields a p-value and, as is general practice in Neuroscience, one can define a threshold value and obtain a map of "significant" connections (the "green-black diagrams" mentioned by the Reviewer) [1]. Does that mean that we consider functional connectivity a binary quantity? Certainly not. First, these "binary" connectivity patterns will depend on the set threshold. Different thresholds will, in general, produce (slightly) different "significant" connectivity patterns. In other words, whether we consider two units as "functionally connected" might depend on the specific value that we choose for the threshold. Second, in addition to a connectivity pattern, our G-ETM and G-ETMV Granger causality methods produce, as noted by Reviewer 1 in his point A3, also a set of interaction functions and exogenous components that give a more nuanced picture of the estimated functional connections.

In closing, Reviewer 1 raises the point of whether a ground-truth functional connectivity for a real physical system can be experimentally assessed at all. Also this question is highly non-trivial and it has deep implications. The Reviewer seems to suggest that the answer is, in general, negative. His point, if we understood it correctly, is that, for a real physical system, we can only operationally measure functional connectivity by means of a model. Different models will give, in general, different patterns of connectivity and which one we "choose" depends, in a Bayesian framework, on the posterior distribution of the likelihood of the model parameters. This is an interesting suggestion that, in the resubmitted manuscript, led us to change the description of the results shown in Fig. 7. Instead of referring to a "ground-truth" functional connectivity, we now discuss those results in terms of two alternative models: one that incorporates exogenous inputs, namely our G-ETM model, and one that does not (the model from

[1] Of course, bearing in mind all the caveats that the definition of arbitrary thresholds for p-values has in modern statistics.

Kim et al.), with the former having a higher likelihood to be appropriate given the characteristics of the analyzed spike trains. The relevant text is on pags. 11 and 12 of our revised manuscript.

2 Stationarity

In the Discussion and around lines 475ff. the authors refer to nonstationarity as the reason why an extension of Kim et al.'s model is needed. I do not think that stationarity is an issue for any of the models (2), (4), or (6). As I see it, time-stationarity is needed for a well-defined frequency analysis, and therefore only necessary for frequency-based methods (such as Granger's initial one, Geweke, and others), not for the methods of the present manuscript. – But I have not carefully analysed this mathematically, so I can be wrong.

Regarding the statement on line 367: "The jointly stationarity assumption gives Granger causality several appealing characteristics [ref. to Seth et al.]", I believe the authors are referring to Seth et al.'s statement "Given these assumptions, G-causality has several attractive properties". Note, however, that the given in that statement does not mean because of, but in the context of – or even notwithstanding. So their meaning is almost opposite. The real reason why model (2) needs to be extended is because it does not include quantities that we know are potential causes. That is, it violates the basic Granger-causality assumption that:

$$P(A|B) = P(A|B, C, D, \dots) \text{ for any other quantities } C,\ D,\ \dots \quad \text{(r1)}$$

where B, C, D denote disjoint sets of time-dependent quantities. See the discussion in Granger (1988), especially p. 200, and also Granger & Thomson (1987). Model (2) assumes that no other input quantities besides the activities of the units can influence the system; that is,

$$B := \textit{activities of the units},\ C := \textit{external inputs},\ D := \textit{anything else},$$

$$P(A|B) \neq P(A|B, C) = P(A|B, C, D, \dots) \text{ (r3)}$$

so that requirement (r1) is violated by C. To satisfy it again we must include C with B. Model (4) does this by adding the time-dependent terms α.

In passing I may add that such inclusion could also have been done by adding a real-valued term $R_{0,m}(t)$ representing external input. Such a term would have been externally given if known (this is essentially what is done in Mimica et al. 2018 although they do not care about Granger causality), or inferred in block with the corresponding $\gamma_{i,0,m}$ if unknown; model (4) does the latter.

In this regard, the new statement around lines 96/100 misses the point: It is not that "the union of the events B and C no longer represents all available information", but that C turns out to be relevant, eq. (r3), whereas before it was assumed to be irrelevant, eq. (r2). See again the initial discussion in Granger (1988).

We thank Reviewer 1 for pointing out this inaccuracy from our side. Following his comment we edited our description of the results of Fig.1 to provide a more precise theoretical frame. The relevant text is on pag. 5 of the resubmitted manuscript

With respect to the stationarity issue, the Reviewer is correct in noting that time-defined Granger causality can be, in principle, applied also to non-stationary data. However, this holds only for particular types of non-stationarity for which specific techniques have been developed (see for example the discussion in [2]). In the general case of standard Granger causality with autoregressive models, stationarity of the data is required to ensure that the parameters of the models converge to meaningful results [2].

3 Additional points

- *I recommend that the application to integrate-and-fire units of lines 182ff. be mentioned and even emphasized in the Abstract. Please note that many works in the literature (Averbeck 2009; the book Grün & Rotter 2010 and refs therein; Brette 2015 fig. 2A) report that real neurons in many circumstances or brain areas are not Poissonian, so some readers will disagree with the justification for the modifications mentioned on lines 561-566. It is a pity that results are not reported for the case without modifications; they would have been extremely interesting, no matter the results. But I am not requiring the authors to report them.*
See also point A2 below.

Following the Reviewer's advice, we now explicitly mention in the Abstract the application of our G-ETM method to spike trains generated by means of a network of integrate-and-fire units.

In addition, Reviewer 1 is correct in stating that violations of the Poisson assumption have been occasionally reported in some experimental studies. To acknowledge this fact, we toned down the motivation for our choice of simulating Poisson integrate-and-fire units. The revised text is on pag. 19 of the revised manuscript.

With respect to the last part of the Reviewer's comment, we would like to emphasize that, in our paper, we do present results obtained without modifying the parameters of the integrate-and-fire units. Indeed, in Fig. 10 we show simulations in which, the threshold of our integrate-and-fire

units was fixed (as it is normally the case with simulated integrate-and-fire units). Such units produce, as expected, extremely regular spike trains.

• *In Fig. 4 and line 157, please make clear according to which distribution the "random" generation occurs.*

In describing results in Fig. 4, we now state that the distribution is uniform. The relevant text is on pag. 7 of the revised manuscript.

• $A_{i,p}$ *on lines 313-325 should be* β*?* • *Please note that "Montecarlo" is spelled 'Monte Carlo' (according to Oxford, Cambridge, Merriam-Webster). There are some strange commas across the paper, e.g. "If," line 95, "emphasized, that" line 244.*

We corrected all these typos.

In addition to useful comments and suggestions, Reviewer 1 also shared some personal concerns. While they were not meant to be suggestions for amendments, they were particularly insightful and we address them below. As for the points above, we use *italic text* to report verbatim the Reviewer's text.

A1 "Validation"

A parametric model such as eq. (2), (4), or (6) is a manifold of probability distributions within a larger space, more precisely a simplex (in this case a simplex of 'point processes'). By definition, a long run of samples from a specific distribution chosen from the model defines a unique point in that manifold. And a fitting/optimization algorithm must obviously recover that unique point in that manifold.

A model such as eq. (2) is a proper submanifold within the manifold of the more general model eq. (4). They are nested models. A fitting algorithm for the submodel can therefore never recover a point generated by a distribution chosen from the more general model, unless that distribution is itself an instance of the submodel.

These are mathematical tautologies.

Numerical implementations of the random generator or of the fitting procedure can of course go wrong: they may have bugs or converge too slowly for practical purposes. The mathematical tautologies above – which must be satisfied by the numerical implementation – are therefore used to check for bugs and convergence.

Figures 1, 2, 3, 4, 8 and related discussion in the manuscript validate that the numerical algorithms used by the authors satisfy these basic requirements. Therefore, if these checks failed they would invalidate the optimization algorithm (or the random generator), they would not invalidate the model. So the statements on line 178 and line 326, "the model is validated. . . ", are incorrect. Any model perfectly fits itself. Points deliberately chosen in the manifold are obviously in the manifold.

Now if the purpose of the authors is to make their numerical optimization code publicly available for use, then it is important that they show that their code is bug-free and converges, and those figures and their discussion are justly emphasized.

But this mathematically guaranteed convergence tells us nothing whatsoever on how a model fares with real data, that is, with points outside the model's manifold.

So, if the purpose of the authors is not to share a new numerical optimization algorithm, but to propose the use of the models (4) & (6), then Figs 1, 2, 3, 4, 8 and their discussion are somewhat superfluous. They could not have been otherwise, no matter the model. Of course the readers

expect the numerics to be correct; it is nice that the authors show that they are correct, but this could have been done in supplementary material.

In this case a reader will wonder "why are the authors emphasizing this tautology?". Some readers may suspect that the authors are trying to deceived them; other readers may get the impression that the authors do not have a full statistical grasp of what they are doing. These possibilities would be detrimental to the model and to the manuscript.

(It's as if you wanted to convince me that you're really good at finding lost keys, and as a proof you put a key in your pocket and then tell me "Now I'll find the key", and take it out of your pocket. Well, no surprise, you put the key there yourself! Are you making fun of me? This doesn't tell me anything about your skills in finding lost keys.)

Again, I think that the proposed approach and model are useful. Therefore it would be sad if any readers of this manuscript felt such kind of misgivings.

Reviewer 1 correctly notices that it is a mathematical tautology that a model fits data generated according to its assumptions. Testing a model on such data sets does not validate the mathematical definition of that model (which is automatically "validated" by the assumptions being met), but its implementation.

Before directly addressing this comment from Reviewer 1 we would like to emphasize two points concerning our study.

First, the main goal of our paper is to show that, under common experimental conditions (i.e. exogenous modulations of the firing rates), our G-ETM and G-ETMV methods work where the previous method by Kim et al. fails. For this reason, throughout our paper we never present the results obtained with the G-ETM or G-ETMV method in "isolation" (with the only exception of Fig. 2), but we always present a comparison between two methods (either G-ETMwith Kim et al.'s method or G-ETM with G-ETMV). In this manner, we can show that the introduction of our new models is not only theoretically justified but it is also of practical relevance. Had, for example, Kim et al.'s method been robust to exogenous modulations of the firing rates, our G-ETM method, although (tautologically) fitting the data, would have been of limited practical relevance. Our comparison of the two methods, shows that this is not the case. In other words, while it is a tautology that G-ETM and G-ETMV fit data generated according to their assumption (provided of course that they are correctly implemented), the non-tautological results of Figs. 3, 4, 5, 8 and 9 are that (1) Kim et al.'s method fail by producing a high number of false positive functional connections and (2) when G-ETM is applied to spike trains containing correlated trial-by-trial variability it fails in a relatively "mild" manner (i.e. a slight increase in the number of false positive functional connections; see Fig. 9).

Second, as also mentioned by the Reviewer, we are presently working to release the code for our G-ETM and G-ETMV methods. To this end, it is also important, that our paper also provides evidence that we implemented them correctly.

That said, Reviewer 1's comment was probably prompted by our potentially incorrect use of the word "validation" when describing the results of Figs. 4, 5 and 9. We thus edited that part of the manuscript. In particular, (1) we now present results in those two figures as a comparison between methods rather than as a "validation" of a method and (2) we use the word "validation" only when referring to our models' implementation (as opposed to their mathematical definition).

A2 "Robustness and applications"

The interesting and non-tautological part, instead, is the application to real spike-train data (Fig. 7). The interpretation and discussion of the results, around lines 275-289, is also very interesting. It is a pity that it is left to so late in the manuscript.

The statement "Although in this case we do no have the ground-truth connectivity pattern, quantitative simulations reported in Figs. 3 and 4 strongly suggest that these additional causal connections are likely false positives" is just wishful thinking, however. For two reasons. First, because we are speaking about functional, not anatomical, connectivity. Is there any "ground truth" for it? See points 1 and A3. Second, because the simulations of Figs 3 & 4 were generated by the general model itself: as discussed in point A1 they don't prove anything about model reliability or generalizability.

What is interesting, instead, are the conditional inferences that we can make by comparing the two models: (I) If there are no exogenous influences, then the sub-model (2) - which would be the most appropriate in this case - indicates some specific internal functional influences, for example from source 6 to target 10. (II) If there are exogenous influences, then the general model (4) - which would be the most appropriate in this case - indicates that no such functional influences occur. In my opinion this is an interesting inference, even if it has a conditional character. In the present case we can consider case (I) to be unlikely (but not impossible) for neurobiological reasons.

Also very interesting is the new application to the artificial network of integrate-and-fire neurons. It would have been interesting to see the inferred functional influences also in this case, similarly to Fig. 7A-B. I am not sure whether Fig. 5 provides any interesting information: again the issue is the difference between anatomical and functional connectivity; see point A3 below.

As I said before, for these simulations it is a pity that the authors made the (unrealistic) modifications described on lines 561-566. It would have been interesting to see the results with and without the modifications.

Finally, also the application of Fig. 10 is extremely interesting. What's funny is that the authors interpret it as an erroneous conclusion on the model's part, whereas I actually suspect that the model did not perform so badly after all. My feeling is that a full (Bayesian) probabilistic analysis would reveal that the model was, so to speak, "undecided" in assigning the correlations to exogenous or to endogenous influences, and that the latter prevailed only by little. This "indecision" could appear as a bimodality in the posterior distribution of the model's parameters, with the second mode (endogenous interpretation) only slightly higher than the first (exogenous interpretation). That is, the curves of Fig. 10C-D could have large error bars around them, actually representing a bimodal distribution in the y-direction. The second mode would be centred on values with small interactions and smaller exogenous components.

This would be a very interesting result and a case in favour of, not against, the model. Unfortunately p-values and significance tests typically miss such important inferences (which is why the American Statistical Association is discouraging their use).

Obviously the measures of "influence" mentioned so far are conditional on the assumptions of the model, but such kinds of assumptions are almost always inevitable. This is related to point A3 below.

We agree with Reviewer 1 that, in practical cases, it might be problematic to define a "ground-truth" functional connectivity and we edited our manuscript to avoid any reference to it in discussing the results of Fig. 7. Instead, we now discuss those results as the "competition" between two models (G-ETM and Kim et al.'s method) of which the former is more likely to be correct (see also our reply to Point 1 above).

We do however believe that the results of Figs. 3 and 4 are not tautological and they have a bearing on Fig. 7. Here are our arguments for that. Reviewer 1 is correct in stating that it is a tautology that G-ETM performs well in the cases shown in Fig. 3 and 4. This is however not the only information that those figures provide. They also show that Kim et al.'s method performs poorly when exogenous modulations are present. This is a non-tautological result (see also our reply to point A1 above) which, paired with the fact that Kim et al.'s model is nested within G-ETM, as also correctly noticed by the Reviewer, supports, in our view, the suggestion that in cases when the two models disagree and exogenous modulations are present in neuronal responses, as is the case with results in Fig. 7, G-ETM is more likely to be correct.

We also agree with the Reviewer that a full Bayesian analysis would be needed to mathematically prove it (and, more in general, to compute the

posterior likelihood of a given model). This would be a highly non-trivial achievement that goes beyond the scope of our study. In our opinion, Figs. 3 and 4 provide the reader with an intuition for that.

A3 "Functional vs anatomical connectivity and meaning of the model"

As mentioned in point 1, functional and anatomical connectivity are distinct notions. This distinction is important for the evaluation of Granger causality models. And it has important consequences for the judgement of the model proposed in the manuscript.

'Functional connectivity' is not a well-defined notion. Just like 'interaction' or 'uncertainty' it can be defined and measured in different ways, all possibly useful in different applications.

My point of view is that the autoregressive model discussed in the manuscript provides one way to define and measure functional connectivity. This remark is valid for any model used for Granger causality. The original studies, for example, focused on what could be called "linear" Granger causality. The present model measures functional connectivity based on a specific autoregressive picture of a neuron's firing rate. Several models may be useful and comparing their measures is useful too. And none of such models can be, or can be meant to be, a physical model: neurons work in completely different ways, dictated by the laws of electromagnetism and chemistry (see Diaconis et al. 2007 for a brilliant discussion about a similar point).

This is why – in my opinion – the manuscript is focusing too much on unimportant matters such as "validation" – which is not provided by self-applications of the model, and even impossible if a ground truth for functional connectivity is undefined – while leaving almost out the really important and useful parts. The point, for me, is not whether the model gives black-or-white answers that coincide with anatomical properties. The model is useful because it quantifies, from a specific point of view, time-directed correlations. The specific curves of Fig. 7C–D, with their numerical values, are important. I personally do not see functional connectivity or interaction as a 0-or-1 quantity, so to me the green-black diagrams of Fig. 7A are uninteresting. But of course they are interesting for readers with a binary conception of functional connectivity. For this reason I was also interested in the application of Fig. 5 but without the modifications listed on lines 561-566. (In the case of only two units the two kinds of connectivity must perhaps be equated.)

We believe that this is a very valid concern from the Reviewer. As also discussed in addressing Point A1 above, it was probably prompted by our potentially incorrect use of the word "validation" in describing the results of Figs. 4, 5, and 9. To address this concern, in the revised version of our

manuscript, we now present the results of those figures as the comparison of different models and we use the word ”validation” only when referring to the implementation, as opposed to its mathematical definition, of our model.

Furthermore, following the Reviewer’s comment on the distinction between functional and anatomical connectivity (see also our reply to Point 1 above) we changed our description of results in Fig. 7. Specifically, we now never mention the word ”connection” alone, but we always use the words ”functional connection”. We would like to emphasize that, in the previous version of the manuscript, the use of the word ”connection” in isolation was only meant to avoid repetition and not as a synonym for ”anatomical connection”. We apologize for any confusion that this might have generated.

References

[1] Marder E, Bucher D. Understanding circuit dynamics using the stomatogastric nervous system of lobsters and crabs. Annual Review of Physiology. 2007;69:291–316. doi:10.1146/annurev.physiol.69.031905.161516.

[2] Seth AK, Barrett AB, Barnett L. Granger causality analysis in neuroscience and neuroimaging. Journal of Neuroscience. 2015;35(8):3293–3297. doi:10.1523/JNEUROSCI.4399-14.2015.
